# dTRPA1 Modulates Afternoon Peak of Activity of Fruit Flies *Drosophila melanogaster*

**DOI:** 10.1371/journal.pone.0134213

**Published:** 2015-07-30

**Authors:** Antara Das, Todd C. Holmes, Vasu Sheeba

**Affiliations:** 1 Evolutionary and Organismal Biology Unit, Jawaharlal Nehru Centre for Advanced Scientific Research, PB # 6436, Jakkur Post, Bangalore-560 064, India; 2 Neuroscience Unit, Jawaharlal Nehru Centre for Advanced Scientific Research, PB # 6436, Jakkur Post, Bangalore-560 064, India; 3 Department of Physiology and Biophysics, University of California, Irvine, D340, Medical Sciences 1, Irvine, California, 92697–4560, United States of America; McGill University, CANADA

## Abstract

Daily rhythms in Drosophila under semi-natural conditions (or SN) have received much recent attention. One of the striking differences in the behaviour of wild type flies under SN is the presence of an additional peak of activity in the middle of the day. This is referred to as the afternoon peak (A-peak) and is absent under standard laboratory regimes using gated light and temperature cues. Although previous reports identified the physical factors that contribute towards the A-peak there is no evidence for underlying molecular mechanisms or pathways that control A-peak. We report that the A-peak is mediated by thermosensitive dTRPA1 (**d**rosophila **T**ransient **R**eceptor **P**otential- A1) ion channels as this peak is absent in dTRPA1 null mutants. Further, when natural cycles of light and temperature are simulated in the lab, we find that the amplitude of the A-peak is dTRPA1-dependent. Although a few circadian neurons express dTRPA1, we show that modulation of A-peak is primarily influenced by non-CRY dTRPA1 expressing neurons. Hence, we propose that A-peak of activity observed under SN is a temperature sensitive response in flies that is elicited through dTRPA1 receptor signalling.

## Introduction

During mid-day, high temperature, low humidity and desiccation present considerable challenges to insect survival. A combination of sensory responses modulated by circadian clock-driven processes allow fruit flies—*Drosophila melanogaster* to coordinate their physiological and behavioural processes to the cyclic external environment [1,2]. While light-driven responses in *Drosophila* are better understood [1,2], how daily thermal cycles influence rhythmic behaviour is less clear [2]. Previous studies have shown that thermal cycles entrain circadian rhythms in *Drosophila* [3–9]. In recent years, behavioural responses of flies under semi-natural conditions have garnered much attention following the publication of a landmark paper by the Kyriacou and Costa groups [10]. In nature, organisms receive multiple time cues or “zeitgebers” in the form of continuously changing environmental variable such as light, temperature, humidity etc. [10–12] in contrast to laboratory regimes which usually provide gated rectangular cycles of light and/or temperature. Under semi-natural conditions (SN), wild type flies exhibit an additional peak of activity during the day, the afternoon or A-peak which previous studies have suggested to be a stress response to high temperature and low humidity conditions during noon [10–12]. The first study to observe the occurrence of A-peak under SN conditions [10] suggested that A-peak was circadian clock driven and also proposed that A-peak was a “stress/escape” response of flies to high temperatures. Similar observations were made by another group of researchers and they posit that although circadian clock may inhibit mid-day activity because it is “unproductive” for the flies, extremely high temperatures elicits A-peak under “life threatening” environments [12]. Another study has suggested that A-peak is circadian clock-independent and elicited due to flies seeking shade within the recording monitors during extremely hot afternoons [11]. More recently it was shown that very high light intensity under SN can also elicit A-peak in several wild caught species of *Drosophila* [13], and that high mid-day temperatures simulated in the lab can induce A-peak even under constant light which is believed to disrupt the circadian clock [14]. All of the above studies suggested that temperature has a crucial role in modulating the occurrence of A-peak under SN. We examined the possible role of an important class of thermoreceptor, the warmth activated ion channel dTRPA1 in eliciting the A-peak under SN.

The molecular and neural circuits that drive behavioural responses to daily cycles of temperature in fruit flies *Drosophila melanogaster* are not well understood. *Drosophila* possess several thermoreceptors [15–18] including a pair of neurons in each brain hemisphere just below the antennae called the anterior cells (AC), which express thermosensitive ion channels dTRPA1 (drosophila Transient Receptor Potential A1) [19]. dTRPA1 has been implcated both in chemical nociception [20] and thermal nociception [21] and it’s role in thermosensation has been studied in great detail by several groups [19,22–27]. Further, dTRPA1 ion channels are known to function at lower temperature 18–19°C also via PLC mediated signalling in larval system [23]. In adults, neurons expressing dTRPA1 ion channels respond to thermal stimuli at two distinct temperatures (25°C and 27°C) [26], similar to the activation range of the channel obtained from whole cell recordings in a heterologous system (24–29°C) [28]. Since these temperatures fall in the range that the adult flies are likely to encounter in nature [13] and because dTRPA1 has wide expression in the adult fly brain, we asked whether dTRPA1 modulates the daily rhythm of activity/rest in *D*. *melanogaster* in response to temperature cycles under SN.

A previous study has shown that intensity of light can modify phase and amplitude of activity peaks under SN [11]. To understand the combinatorial effects of light and temperature on the activity pattern in flies that lacked dTRPA1 function, we recorded the activity of flies under SN, thereby exposing them to an environment highly enriched in terms of time cues compared to the relatively impoverished environmental cues under standard laboratory conditions. We further studied the activity/rest rhythms of flies under controlled simulated SN conditions in the laboratory with gradually changing light and temperature regimes. Here we report the results of our studies using a dTRPA1 driver with restricted pattern of expression, created by Hamada and colleagues; *dTRPA1* ^*SH*^ *-GAL4* [19]. Our results show that *Drosophila* TRPA1 mediates behavioural responses to varying temperature conditions. We demonstrate that under complex semi-natural conditions of fluctuating light, humidity and relatively high temperature maxima, dTRPA1 mediates the afternoon peak of activity. Although previous studies posit that the A-peak reflects escape responses of flies to high temperature conditions [12], the underlying molecular mechanisms that control the A-peak remain unknown. We find that *dTRPA1* null mutants do not exhibit the A-peak and that its amplitude is a function of dTRPA1 expression levels. Dependence of the A-peak on dTRPA1 was further confirmed by studies in the laboratory under controlled simulated natural light and temperature conditions. Our studies reveal a receptor-mediated signalling pathway that adaptively regulates locomotor activity in response to temperature. This exemplifies yet another of the diverse sensory functions of the evolutionarily conserved TRP family of ion channels [29].

## Methods

### Fly strains

All genotypes were reared on standard cornmeal medium under 12:12 hr LD and 25°C. *dTRPA1* ^*SH*^ *-GAL4* [19] driver targets a subset of dTRPA1 expressing neurons (~30 cells) in the adult fly brain. *TRPA1* ^*KI-GAL4*^ [30] has a *GAL4* gene inserted in dTRPA1 promoter region making it a dTRPA1 null but can also drive expression under the dTRPA1 promoter. *TRPA1* ^*KI-GAL4*^ has a much broader target area (~70 cells) with innervations near the fan-shaped body. *Pdf-GAL4* [31] and *cry-GAL4-39* [32] drivers were used to target subsets of circadian neurons. Thermosensor dTRPA1 channel was over-expressed using *UAS-dTRPA1* [25] under *dTRPA1* ^*SH*^ *-GAL4* driver for heat dependent activation of neurons. *UAS-hid* was used for ablation of neuronal subsets (provided by Michael Rosbash, Brandeis). Null mutants used were *dTRPA1* ^*ins*^ (provided by Paul Garrity, Brandeis University) and *TRPA1* ^*KI-GAL4*^ (donated by Youngseok Lee, Kookmin University). dTrpA1 isoforms-*UAS dTRPA1-A* and *UAS-dTRPA1-B* were provided by Paul Garrity and Dan Tracey. Here we use the isoform nomenclature developed by the Montell [33] and Tracey labs [27].

### Behavioural assays

All recordings under semi natural conditions (SN) were carried out in DAM2 monitors in an outdoor enclosure as described previously [11]. Environmental factors—light, temperature and humidity, were simultaneously monitored using DEnM environmental monitors (Trikinetics). To record the activity of flies under DD+SN, DAM2 monitors were placed in light-tight metal boxes kept in the outdoor enclosure. The boxes were fitted with fans and appropriate vents and baffles to enable air circulation and prevent overheating. DEnM monitors were placed inside the boxes to measure light, temperature and humidity in parallel with locomotor activity measured by DAM2 monitors. To mimic the gradual light and temperature cycles of SN in the laboratory, light intensity and temperature were increased in a step-wise manner either alone or in combination. The details of light and temperature conditions of simulated SN regimes are given in S1 Table. All laboratory based protocols involving simulated light and/or temperature were carried out in incubators (Sanyo, Japan and Percival Drosophila chambers, USA). Temperature and light steps were applied using available programs of the respective incubators. DEnM placed within the incubators recorded changes in light, temperature and humidity throughout the experiments. Activity counts in 15 min intervals of individual male flies across at least 5 days were first averaged, following which mean activity counts were averaged across flies of a given genotype and plotted against time of day. Whether a particular genotype exhibited A-peak was determined using visual estimation described previously [14]. Briefly, a fly was considered as exhibiting an A-peak in SN when there was a gradual increase in activity leading to a peak during mid-day (between 11:00 hrs to 16:00 hrs). A-peak estimation was carried out for each fly from its 15 min binned profiles for a single day. A given genotype was considered to exhibit A-peak only when at least 25% of flies displayed A-peak. Proportion of flies showing A-peak was determined by the above method for each day and L_max_ and T_max_ was tabulated using data recorded in DEnM monitors. Data from multiple experiments were pooled to perform Spearman rank order correlation analysis on proportion of flies displaying A-peak each day with their corresponding values of L_max_ or T_max_ using STATISTICA. Mean activity profiles averaged across 5 days for an individual fly were considered for estimating proportion of flies showing A-peak in laboratory simulated regimes. To estimate percentage activity (A-peak) during 1 hr of T_max_ at 32°C, first activity data from individual flies obtained in 15 min bins were normalised to sum of activity across 24 hrs following which the profiles were averaged across 5 days. Thereafter, data across one hour of T_max_ (when temperature was 32°C) was binned to estimate percentage A-activity. Significant differences among genotypes for the A-peak amplitude were determined by one-way-ANOVA on arc-sine transformed data, followed by Tukey’s HSD, *p* < 0.05. Phase of E-peak was estimated subjectively such that the activity data of individual flies averaged across days showed a gradual rise in activity culminating in a peak. E-peak phase of individual flies were averaged to obtain mean E-peak phase for a given genotype. Nonparametric Kruskal-Wallis ANOVA by ranks, followed by multiple comparisons (*p* < 0.05) was performed to compare E-peak phase values among genotypes.

### Immunocytochemistry

Immunocytochemistry was performed as described previously [34] on brains of 2-3-day old adult virgin male flies. Primary antibodies used were chicken anti-GFP (1:1000, Molecular Probes), rabbit anti-PER (1:10,000, donated by Ralf Stanewsky) and mouse anti-PDF (1:5000, DSHB). Secondary antibodies used were Alexa 488 (goat anti-chicken at 1:1500, Molecular Probes), Alexa 546 (goat anti-rabbit at 1:3000, Invitrogen), Alexa 546 (goat anti-mouse at 1:3000, Invitrogen) and Alexa 633 (goat anti-rabbit at 1:3000, Invitrogen). Repression of GAL4 driven expression using *cry-GAL80* was confirmed by GFP expression studies first using Zeiss epifluorescence microscope. Representative specimens were also imaged under Zeiss LSM 700 and Zeiss 510 META confocal microscopes. Images were assembled and brightness and contrast adjusted using ZEN 2011 software (S1A Fig). Ablation of most dTRPA1^+^ neurons by expressing *UAS-hid* was confirmed as the lack of GFP expression thermosensory AC neurons in six out of seven adult fly brains examined using Zeiss epifluorescence microscope (one brain showed a single GFP^+^ cell) (S1B Fig). Confocal microscopy revealed the presence of 2–3 weakly stained cells in the dorsal protocerebrum.

## Results

### dTRPA1 is necessary for the A-peak in semi-natural conditions

**Fig 1:**
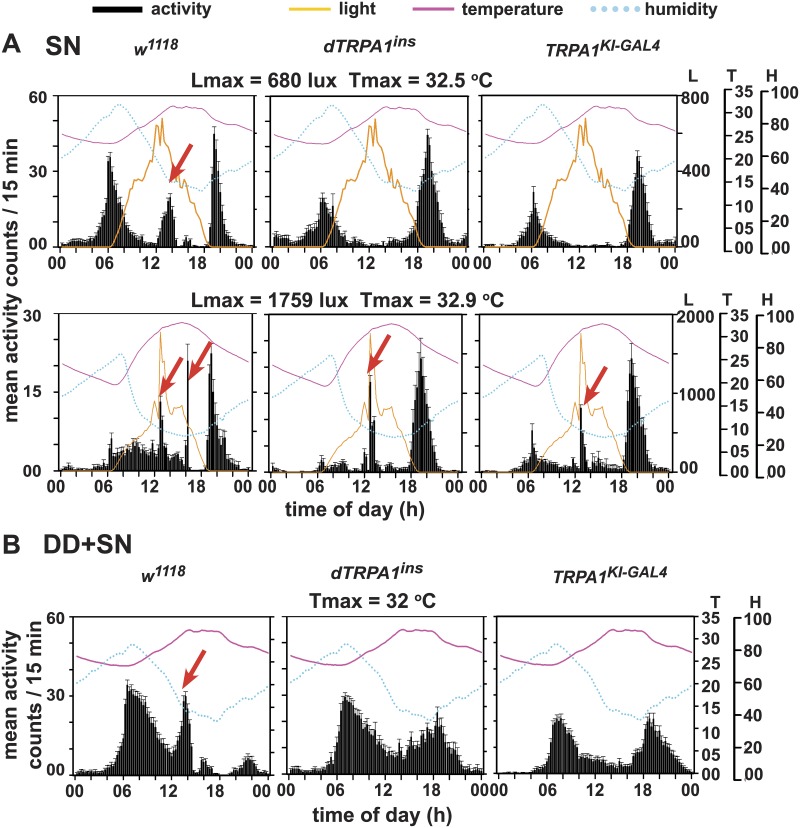
Under semi-natural conditions the mid-day A-peak depends upon dTRPA1. (A) Average activity profiles of wildtype (*w* ^*1118*^) and two dTRPA1 null mutant lines (*dTRPA1* ^*ins*^ and *TRPA1* ^*KI-GAL4*^) under SN conditions with different light intensities. Under low light and high temperature, *w* ^*1118*^ show three peaks of activity with distinct A-peak (arrow) whereas mutant flies display bimodal activity lacking the A-peak. Under high light and high temperature, *w* ^*1118*^ show two distinct peaks in the afternoon- one coinciding with L_max_ and another with T_max_ (arrows). Interestingly, dTRPA1 nulls also show an A-peak corresponding to L_max_ (arrow) but not with T_max_. (B) In DD+SN also, *w* ^*1118*^ depict distinct A-peak (arrow) whereas dTRPA1 null flies do not. Error bars are SEM. Three axes on the right represent environmental factors—light (L-lux), temperature (T-degree Celsius) and relative humidity (H-percentage). Arrows indicate A-peak displayed by more than 25% flies.

Recent studies show that wild type flies exhibit an additional activity peak during mid-day besides the canonical morning and evening peaks under SN [10–12]. Temperature has been shown to be a factor that elicits the A-peak [10,13,14]. We studied the behaviour of flies lacking dTRPA1, a thermosensitive ion channel that has been reported to become activated within the range of 24–29°C [28]. We recorded locomotor activity of flies under semi-natural conditions (labelled SN on the Figures) using the same outdoor enclosure and method as previously described [11]. It has been proposed that under warm conditions in nature, wild type flies suppress their afternoon activity but relieve such inhibition resulting in an A-peak only when average daytime temperatures are high [10,12]. Clock mutants enhance their activity in response to warm daytime temperatures at a much lower range, thus exhibiting ‘unproductive’ afternoon activity [12]. We confirm that wild type (*w* ^*1118*^) flies exhibit three peaks of activity in multiple independent experiments (two of which are described here). When the maximum light intensity (L_max_) reached was either moderate (680 lux) or high (1759 lux), with temperature maximum (T_max_) of ~32°C, *w* ^*1118*^ flies exhibit all three peaks- in the morning (M), afternoon (A, marked by red arrow) and evening (E) (Fig 1A, left panels, Table 1). In contrast, most flies (~87%) with a “strong loss of function” mutation (*dTRPA1* ^*ins*^) [19,35] assayed in parallel display a bimodal activity pattern with no A-peak (Table 1; Fig 1A, top-middle panel). An independently derived dTRPA1 null mutant [30], *TRPA1* ^*KI-GAL4*^, also does not exhibit an A-peak when temperature is highest and humidity is lowest under SN (Table 1 (~82%); Fig 1A, top-right panel). These results indicate that the A-peak is a dTRPA1-dependent response.

**Table 1:** Percentage of flies from different genotypes exhibiting A-peak across SN and laboratory simulated SN regimes.

Regime	Genotype	N	% flies exhibiting A-peak
**SN—L**_**max**_ **680 lux**	*w* ^*1118*^	30	78
	*dTRPA1* ^*ins*^	15	13.3
	*TrpA1* ^*KI-GAL4*^	14	17.6
**SN—L**_**max**_ **1759 lux**	*w* ^*1118*^	28	84.2 (L_max_ -88%; T_max_ -67%)
	*dTRPA1* ^*ins*^	16	57.8 (L_max_ -91%; T_max_ -13%)
	*TrpA1* ^*KI-GAL4*^	20	70.3(L_max_ -100%; T_max_ -16%)
**DD+SN**	*w* ^*1118*^	28	85.7
	*dTRPA1* ^*ins*^	12	NA
	*TrpA1* ^*KI-GAL4*^	25	NA
**L**_**r**_ **+T**_**r28**_	*w* ^*1118*^	16	6.25
	*dTRPA1* ^*ins*^	23	4.3
**L**_**r**_ **+T**_**r32**_	*w* ^*1118*^	28	100
	*dTRPA1* ^*ins*^	27	14.8
	*TrpA1* ^*KI-GAL4*^	26	3.8
	*dTRPA1* ^*SH*^ *-GAL4/ UAS dTRPA1*	27	100
	*dTRPA1* ^*SH*^ *-GAL4/ +*	23	100
	*UAS dTRPA1/ +*	28	100
**L**_**r**_ **+T**_**r32**_ **(out of phase)**	*w* ^*1118*^	30	100
	*dTRPA1* ^*ins*^	26	11.5
	*dTRPA1* ^*SH*^ *-GAL4/ UAS dTRPA1*	28	100
	*dTRPA1* ^*SH*^ *-GAL4/ +*	29	100
	*UAS dTRPA1/ +*	26	61.5

Under SN—L_max_ 1759 lux, percentage of flies that exhibit A-peak coinciding with L_max_ or T_max_ are indicated in parentheses. NA* Distinct A-peak could not be determined because of high activity exhibited by flies throughout the day. For details regarding genotypes, please see Methods section.

Previous studies suggest that the A-peak is temperature dependent [10,12–14]. The proportion of *w* ^*1118*^ flies that exhibit the A-peak is positively correlated with T_max_ (Spearman’s rank order correlations, *r* = +0.78, *p* < 0.05) but no significant correlation is detectable with L_max_. On the other hand, the small proportion of *dTRPA1* ^*ins*^ flies that do exhibit the A-peak is correlated with L_max_ (*r* = + 0.51, *p* < 0.05) but not with T_max_. Similarly, the small proportion of *TRPA1* ^*KI-GAL4*^ flies that do display the A-peak is positively correlated with L_max_ (Spearman’s rank order correlations, *r* = + 0.56, *p* < 0.05). Proportion of *TRPA1* ^*KI-GAL4*^ flies that exhibit the A-peak is also found to be negatively correlated with T_max_ (Spearman’s rank order correlations, *r* = - 0.61, *p* < 0.05) probably due to a few days with high light intensity but low T_max_.

Both the null mutants, *dTRPA1* ^*ins*^ and *TRPA1* ^*KI-GAL4*^ are sensitive to other environmental factors and exhibit small spikes in their activity throughout the day in response to very bright light similar to *w* ^*1118*^ flies (>1700 lux, Fig 1A, bottom row). However, under very bright light conditions, *w* ^*1118*^ flies show two bouts of intense activity during mid-day—one coinciding with L_max_ and distinct from the second that coincides with T_max_, illustrating that *w* ^*1118*^ flies which have intact photosensitivity and thermosensitivity show two distinct peaks of activity in response to temporally separated L_max_ and T_max_. In contrast, dTRPA1 null flies respond with activity peaks only in response to L_max_ under very bright light conditions and presumably due to compromised thermal sensitivity, do not respond to T_max_. Under high light intensity, activity levels are suppressed for all genotypes compared to low light conditions; even at sunrise although light intensity is not as bright as during mid-day (compare Fig 1A, top vs bottom panels). In addition to light intensity, both temperature and humidity (and possibly other factors) differ between the two experiments under SN. At sunrise, along with high light intensity, humidity is lower by about 20% and temperature is lower by 5°C. Hence, we speculate that perhaps light is not the major modulator of activity during sunrise.

To eliminate the effect of light on the A-peak, we recorded activity of flies in light-tight metal boxes kept in the same outdoor enclosure (henceforth, DD+SN). Under DD+SN, where flies experience daily variation in temperature and humidity but not light, *w* ^*1118*^ flies show a distinct A-peak whereas both null mutants- *dTRPA1* ^*ins*^ and *TRPA1* ^*KI-GAL4*^ do not show the A-peak (Fig 1B, Table 1). In contrast to SN, under DD+SN, only 60% of control *w* ^*1118*^ flies show E-peak, while among the mutants 87% of *dTRPA1* ^*ins*^ and 100% of *TRPA1* ^*KI-GAL4*^ exhibit it (Fig 1B). Further, *w* ^*1118*^ flies exhibit a significantly delayed E-peak compared to *dTRPA1* ^*ins*^ and *TRPA1* ^*KI-GAL4*^ (Kruskal-Wallis ANOVA by ranks followed by multiple comparison). This may be possibly due to compensatory reduction in activity due to excessive activity at noon. We also tested flies wherein dTRPA1 neurons were ablated using a restricted GAL4 driver- *dTRPA1* ^*SH*^ *-GAL4* [19] under SN (S2A Fig) and DD+SN (S2B Fig) where T_max_ reached 32°C. However, in both cases, flies with ablated dTRPA1^+^ neurons (blue curves) and their parental controls (grey and black curves) displayed small A-peaks (S2A and S2B Fig, left panels). We also note that under DD+SN, among the four control genotypes, one line, namely the *UAS-hid* flies exhibit an enhanced E-peak, which could be due to leaky expression of *hid* resulting in loss of cells that influence the E-peak (S2B Fig, left panel). Thus, we find that while complete lack of dTRPA1 renders flies incapable of exhibiting the A-peak (Fig 1), flies with partial reduction in dTRPA1 expression or ablation of neurons targeted by *dTRPA1* ^*SH*^ *-GAL4* do not phenocopy dTRPA1 nulls (*dTRPA1* ^*ins*^ or *TRPA1* ^*KI-GAL4*^).

We reasoned that if the A-peak depends on dTRPA1 expression in the *dTRPA1* ^*SH*^-*GAL4* driven neurons, A-peak would become amplified upon enhanced expression of dTRPA1 in these thermosensory neurons. Upon over-expression of dTRPA1 under *dTRPA1* ^*SH*^-*GAL4* (*dTRPA1* ^*oex*^) flies show A-peak not significantly higher than controls (S2A Fig, right panel). We speculate that the bright light conditions dampened the A-peak expected of *dTRPA1* ^*oex*^ flies. This hypothesis is supported by tests under DD+SN where *dTRPA1* ^*oex*^ flies show a striking enhancement of the amplitude of their A-peak (S2B Fig, right panel, arrow). Increasing the expression level of dTRPA1 is likely to enhance the firing rate of these neurons at temperatures of 25–27°C and higher [19,26], which we propose results in the induction of the A-peak. Over-expression of dTRPA1 also delays E-peak of *dTRPA1* ^*oex*^ flies (S2B Fig, right panel and arrowhead).

### dTRPA1 levels influence the amplitude of A-peak under simulated natural conditions

**Fig 2:**
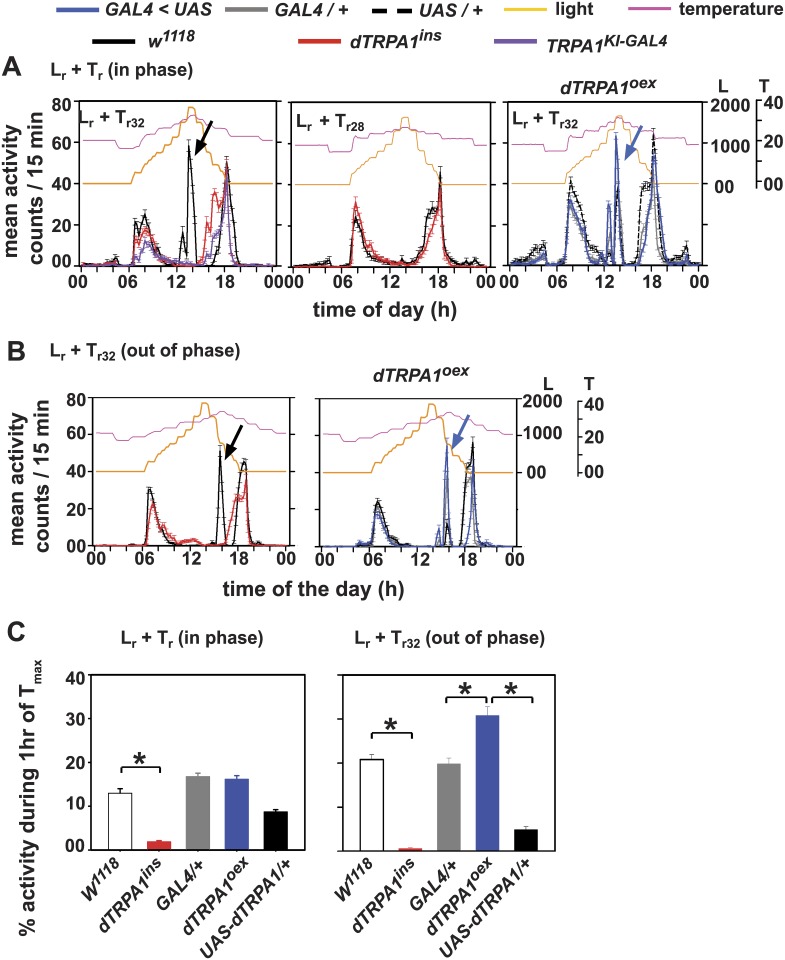
Simulating natural temperature profile in the laboratory generates dTRPA1-dependent A-peak. (A, left) Average activity profiles of flies under L_r_+T_r32_ (L_max_ and T_max_ occur in phase) wherein wild type *w* ^*1118*^ show a prominent A-peak (arrow) but dTRPA1 null flies (*dTRPA1* ^*ins*^- red and *TRPA1* ^*KI-GAL4*^-violet) do not show an A-peak. (A, left) Average activity profiles of flies under L_r_+T_r32_ (L_max_ and T_max_ occur in phase) wherein wild type *w* ^*1118*^ show a prominent A-peak (arrow) but dTRPA1 null flies (*dTRPA1* ^*ins*^- red and *TRPA1* ^*KI-GAL4*^-violet) do not show an A-peak. (A, -middle) Flies under simulated light and temperature protocols with different temperature maxima (L_r_+T_r28_). Neither genotype exhibits an A-peak under L_r_+T_r28_ when T_max_ reached 28°C. (A, right) *dTRPA1* ^*oex*^ flies (blue curves) show A-peak similar to their controls under L_r_+T_r32_. (B, left) Activity/rest profiles of *w* ^*1118*^ depict a distinct A-peak corresponding to T_max_ but not to L_max_ under out of phase L_r_+T_r32_ whereas *dTRPA1* ^*ins*^ flies do not show any peak. (B, right) *dTRPA1* ^*oex*^ flies show an enhanced A-peak compared to controls and the peak coincides with T_max_. All control flies showed an A-peak conjugated with T_max_ like *w* ^*1118*^ flies. (C, left) Percentage activity during 1 hr of T_max_ (32°C) when light and temperature gradually changed (left) in phase and (right) out of phase. Under both regimes, *dTRPA1* ^*ins*^ flies showed significantly reduced activity compared to *w* ^*1118*^. *dTRPA1* ^*oex*^ flies showed higher A-peak activity than controls under out of phase L_r_+T_r32_. All other experimental details are same as in Fig 1.

**Fig 3:**
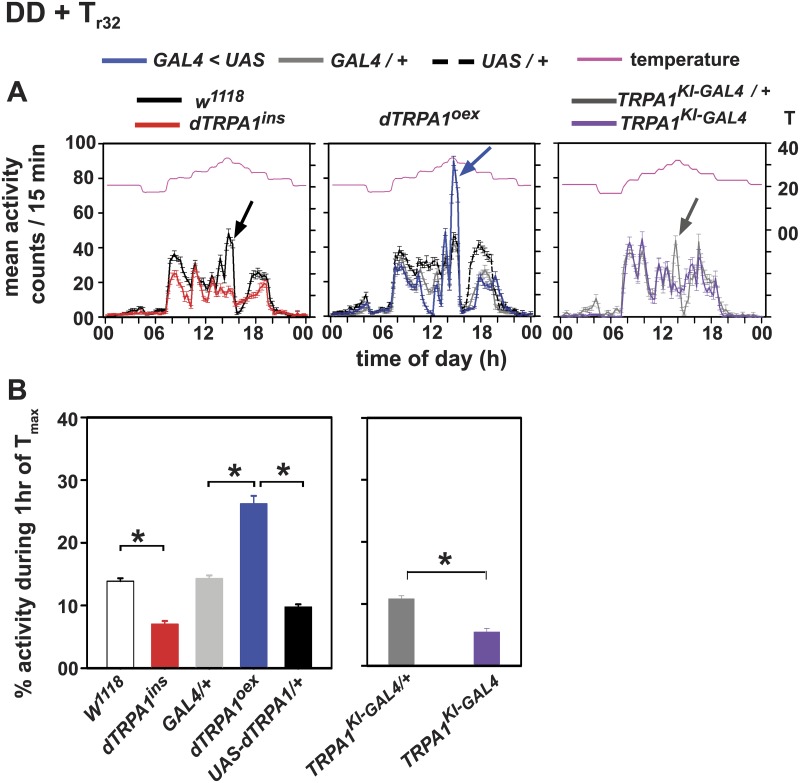
dTRPA1-dependent A-peak can be elicited by gradual temperature cycles even in absence of other time cues. (A) Flies were subjected to simulated temperature protocol under constant darkness (DD+T_r32_). All control genotypes show an A-peak. (Left) *w* ^*1118*^ flies display A-peak coinciding with T_max_ 32°C whereas *dTRPA1* ^*ins*^ flies do not, although the latter responded to temperature changes outside dTRPA1 activation range similar to *w* ^*1118*^. (Middle) *dTRPA1* ^*oex*^ flies show an enhanced A-peak compared to their respective controls. (Right) Heterozygous *TrpA1* ^*KI-GAL4*^ */+* flies (violet curve) show A-peak whereas homozygous *TrpA1* ^*KI-GAL4*^ null flies (grey curve) do not. (B, left) Comparison of percentage activity during 1 hr of T_max_ (32°C). *dTRPA1* ^*ins*^ flies show significantly reduced activity compared to *w* ^*1118*^ and *dTRPA1* ^*oex*^ flies show higher A-peak activity than controls under DD+T_r32_. (B, right) Heterozygous *TrpA1* ^*KI-GAL4*^ */+* flies show higher A-peak activity than *TrpA1* ^*KI-GAL4*^ null flies. All other experimental details are the same as in Figs 1 and 2.

**Fig 4:**
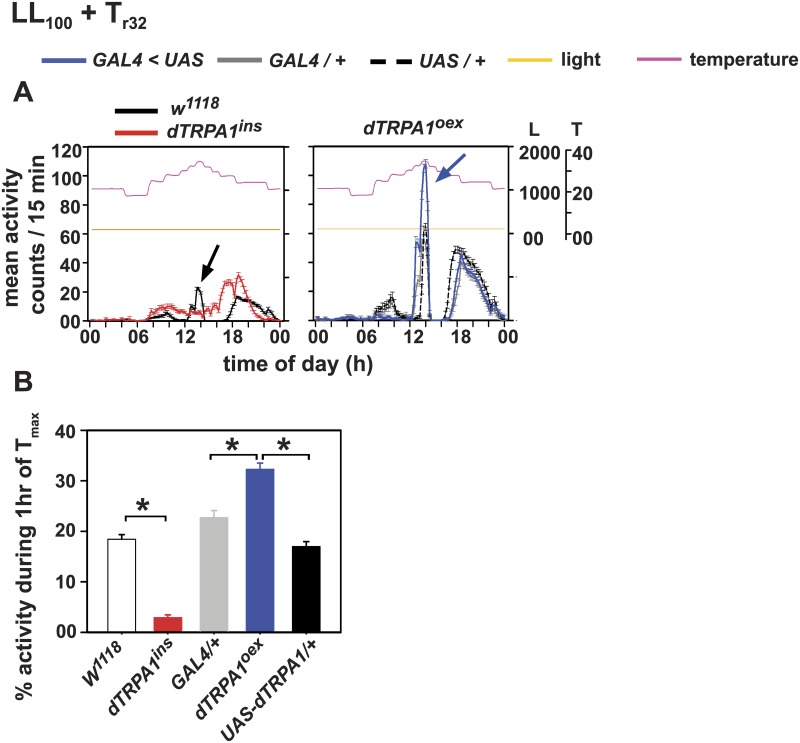
dTRPA1-dependent A-peak can be elicited by gradual temperature cycles under constant light. (A) Average activity profiles of flies in simulated natural temperature cycles under constant light (100 lux) (LL_100_+T_r32_). (Left) *w* ^*1118*^ flies display an A-peak while *dTRPA1* ^*ins*^ flies do not. (Right) *dTRPA1* ^*oex*^ flies show an enhanced A-peak compared to their parental controls. (B) Percentage activity during 1 hr of T_max_ (32°C). *dTRPA1* ^*ins*^ flies showed significantly reduced activity compared to *w* ^*1118*^ and *dTRPA1* ^*oex*^ flies show higher A-peak activity than controls under LL_100_+T_r32_. All other experimental details are the same as in Figs 1 and 2.

**Fig 5:**
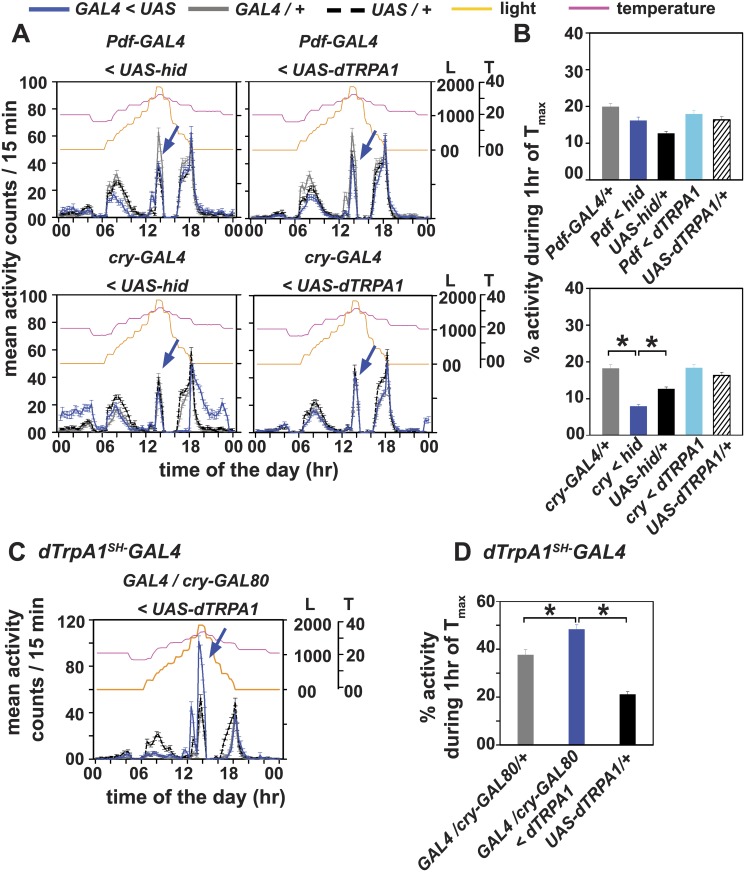
CRY-negative dTRPA1 neurons mediate the enhanced A-peak under gradual light and temperature cycles. (A) Neuronal ablation or dTRPA1 over-expression either under (top) *Pdf-GAL4* or (bottom) *cry-GAL4* does not abolish occurrence of A-peak under L_r_+T_r32_. (B) Comparison of percentage activity during 1 hr of T_max_ (32°C). (B, top) *Pdf-GAL4* mediated ablation or dTRPA1 over-expression elicits A-peak similar to the A-peak amplitude of their respective controls. (B, bottom) Ablation of CRY positive neurons leads to reduction in A-peak activity compared to controls whereas over-expression of dTRPA1 in CRY positive neurons has no effect on A-peak amplitude. (C) Over-expression of dTRPA1 only in non-CRY neurons targeted by *dTRPA1* ^*SH*^ *-GAL4* (*dTRPA1* ^*SH*^ *-GAL4* + *cry-GAL80* < *UAS-dTRPA1*), leads to enhanced A-peak (blue curve, arrow) compared to controls. (D) Comparison of percentage activity also depicts that flies over-expressing dTRPA1 in CRY negative, dTRPA1^+^ neurons show enhanced A-peak compared to controls. All other experimental details same as Figs 1 and 2.

**Fig 6:**
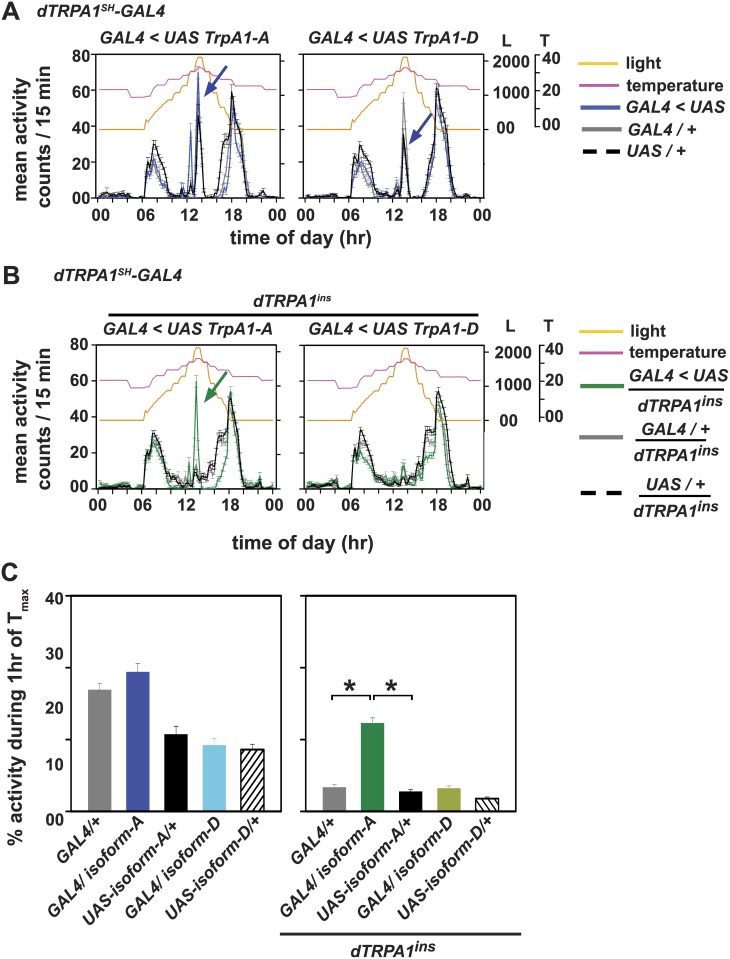
dTRPA1-A isoform rescues the mutant phenotype of dTRPA1 null flies under gradual light and temperature cycles. (A) Overexpression of dTRPA1 isoforms dTRPA1-A or dTRPA1-D under *dTRPA1* ^*SH*^ *-GAL4* is able to elicit A-peak under L_r_+T_r32_. (B) Rescue of dTRPA1 function in *dTRPA1* ^*ins*^ background only with dTRPA1-A but not with dTRPA1-D isoform. (B, left) dTRPA1-A which is known to be active 24–29°C elicits an A-peak similar to *UAS-dTRPA1* mediated A-peak when driven under *dTRPA1* ^*SH*^ *-GAL4*. (B, right) Restoring dTRPA1-D isoform expression in *dTRPA1* ^*ins*^ null background induces an A-peak not different from the parental controls in null background. dTRPA1-D isoform, whose activation threshold is ~34°C, is not able to rescue occurrence of A-peak in mutant background. (C, left) Comparison of percentage activity show that over-expression of either dTRPA1-A or dTRPA1-D isoform does not alter A-peak amplitude compared to their respective controls during 1 hr of T_max_. (C, right) Restoring expression of only dTRPA1-A isoform under *dTRPA1* ^*SH*^ *-GAL4* in *dTRPA1* ^*ins*^ null background is able to elicit A-peak in flies compared to their parental controls in null background which have significantly low levels of A-peak activity. All other experimental details are same as in Figs 1 and 2.

To mimic aspects of natural light and temperature in the laboratory, we exposed flies to gradually changing light intensity and temperature by a series of controlled steps using incubators in the laboratory (Figs 2–6). Since we found that maximum temperature reached in SN was around 32°C, our temperature cycles were calibrated to reach a T_max_ = 32°C (Fig 2A, pink curves). Under such a regime both light and temperature gradually increased and decreased in-phase (L_r_+T_r32_—in-phase). Wild type (*w* ^*1118*^) flies exhibit the A-peak under L_r_+T_r32_ but neither *dTRPA1* ^*ins*^ nor *TRPA1* ^*KI-GAL4*^ flies exhibit the A-peak under such simulated conditions in the laboratory (Fig 2A top-left panel, S3A Fig, Table 1). Henceforth, only *dTRPA1* ^*ins*^ null flies are shown for comparison with wild type flies under different regimes unless specified. We subsequently subjected same genotypes to a similar regime where light and temperature varied gradually except that the T_max_ did not increase beyond 28°C (L_r_+T_r28_) (Fig 2A, top-right panel). Interestingly under this regime, *w* ^*1118*^ flies also did not exhibit A-peak similar to *dTRPA1* ^*ins*^ null mutant (Fig 2A top-right panel, S3A Fig, Table 1) suggesting that T_max_ beyond 28°C is required to elicit A-peak. Next, we tested *dTRPA1* ^*oex*^ flies that have increased levels of dTRPA1 under the simulated L_r_+T_r32_ in the laboratory; a regime in which wild type flies exhibited a clear A-peak (Fig 2A, bottom panels). Consistent with our hypothesis, overexpression of dTRPA1 resulted in an A-peak coinciding with temperature steps above 28°C (Fig 2A, right; 2C, left and S3A Fig). Similar to *w* ^*1118*^ flies, *dTRPA1* ^*oex*^ flies do not exhibit A-peak under L_r_+T_r28_ and resemble their respective controls (data not shown). Thus, when gradually changing light and temperature cycles were provided with T_max_ of 32°C, null mutation of dTRPA1 prevented the induction of A-peak whereas over-expression of dTRPA1 in neurons targeted by the *dTRPA1* ^*SH*^ *-GAL4* driver moderately increases mid-day activity. Hence we propose that the A-peak seen under laboratory simulated natural conditions is elicited via dTRPA1.

In nature, light intensity often reaches its peak prior to temperature. We have seen previously that under SN, wild type flies exhibit two peaks during the day- one coinciding with L_max_ and the other T_max_ (see Fig 1A). To further dissociate the potential effects of light and temperature, we subjected flies to a regime where light intensity peaked (L_max_ ~ 1700 lux) 3 hr prior to temperature peak (T_max_ = 32°C). All control genotypes show A-peak coinciding with T_max_ (Fig 2B black, grey curves). However, the laboratory simulated regime could not evoke the corresponding peak with L_max_, possibly because we do not adequately simulate the light intensity and other spectral qualities of light present in nature. As expected, dTRPA1 null mutant did not show A-peak coinciding with either L_max_ or T_max_ (Fig 2B, red curve, left panel). Under this regime also *dTRPA1* ^*oex*^ flies show an enhanced A-peak higher than both parental control flies (one way ANOVA followed by Tukey’s HSD, *F*_(2,80)_ = 112.83, *p* < 0.05) which coincided with temperature steps above 28°C and not with L_max_ (Fig 2B and 2C right panels). Thus, we show that the temperature dependent A-peak is elicited by dTRPA1 expression levels. In a separate experiment, we first subjected the flies to LD (12:12) for three days followed by LD in combination with gradual temperature cycles (LD+T_r32_) such that the T_max_ either occurred 3hr before Lights-OFF (LD+T_r1_) or 3hr after Lights-ON (LD+T_r2_) (S3B Fig). *w* ^*1118*^ flies exhibit an A-peak coinciding with T_max_ irrespective of the time of T_max_ whereas *dTRPA1* ^*ins*^ flies do not display an A-peak in response to T_max_ (S3B Fig). Thus we confirm that the A-peak seen under SN is a temperature—dependent behaviour modulated by dTRPA1 and does not appear to be directly circadian clock dependent.

### A-peak can be elicited by gradual temperature cycles in absence of other time cues

To determine if dTRPA1 activation by temperature is sufficient to elicit the A-peak in the absence of all other time-cues that are normally present in nature, we simulated natural temperature cycles in the laboratory under constant darkness (DD+T_r32_) such that temperature is the only gradually changing variable. Under DD+T_r32_, all other genotypes show the A-peak while *dTRPA1* ^*ins*^ flies do not (Fig 3A and 2B, left panels, red). Further, under DD+T_r_, all flies show small bouts of activity at every step-increase in temperature, with the most dramatic increase in response to the 32°C step, however *dTRPA1* ^*ins*^ flies respond to the increasing steps early in the day, while steps coinciding 30°C and 32°C have very small responses suggesting the critical need for dTRPA1 channels for this behaviour. *dTRPA1* ^*oex*^ flies show enhanced activity compared to their respective parental controls (Fig 3A, middle panel; Fig 3B, left panel). We further corroborated these results by comparing *TrpA1* ^*KI-GAL4*^ null flies with their heterozygotes. Heterozygous *TrpA1* ^*KI-GAL4/+*^ flies display A-peak like wild type control flies (Fig 3A, right panel; 3B, right panel and S2 Table) whereas homozygous null mutants do not exhibit A-peak suggesting the knock-in mutation is recessive. These results are similar to those obtained under DD+SN and together demonstrate that gradually changing temperature cycles which reach above 28°C are necessary and sufficient to induce the dTRPA1 mediated A-peak. Thus, activation of dTRPA1^+^ neurons causes enhancement of locomotor activity when temperature gradually rises towards 30°C in the middle of the day (Figs 1–3). Surprisingly, in contrast to acute activation by over-expression of dTRPA1 (which would cause activation only under warm temperatures of the T_r_), *UAS-NaChBac* [34,36] induced hyperexcitation which is chronic, did not have a similar effect. Instead, A-peak amplitude is similar to controls, while the E-peak is delayed (S4 Fig). Thus the amplitude of the A-peak appears to depend on whether the activation of these neurons is chronic or acute.

Under constant light (LL), locomotor activity/rest behaviour of wild type flies is arrhythmic [37–41] but rectangular temperature cycles can entrain the circadian clock even in LL [4,18]. To determine if the dTRPA1 mediated A-peak can be elicited under LL conditions, we provided gradually changing temperature cycles with T_max_ = 32°C (LL+T_r32_). Although overall activity levels are low, wild type (*w* ^*1118*^ *)* flies exhibit a mid-day peak coinciding with temperatures above 28°C (Fig 4A, left panel; Fig 4B). The null mutant, *dTRPA1* ^*ins*^ flies respond to temperature step-ups below the range of dTRPA1 channel activation with small increases in activity but show no A-peak (Fig 4A, left panel; Fig 4B). As expected, *dTRPA1* ^*oex*^ flies (blue curve) show a large and significant increase in activity levels compared to the controls during 1hr interval when maximum temperature is reached (T_max_) (Fig 4A, right panel; Fig 4B). Thus, the expression levels of dTRPA1 modulates the amplitude of temperature-dependent A-peak under temperature cycles even in constant light conditions which otherwise disables molecular circadian clock cycling.

### dTRPA1 neurons distinct from cells overlapping with cryptochrome expressing neurons modulate A-peak under gradual light and temperature cycles

While our results thus far suggest independence of the A-peak from the circadian clock, previous reports have suggested that the phasing of onset of afternoon activity is modulated by circadian clocks [10,12]. The expression pattern of the *dTRPA1* ^*SH*^ *-GAL4* driver has been shown to overlap with circadian clock neurons-fifth s-LN_v_, three CRY-positive LN_d_, and one DN_1a_ [42]. This finding has some agreement with a recent study that reports a much broader overlap, with up to three cells in each of the circadian neuronal subgroups using a driver line that has *GAL4* knocked-into dTRPA1 promoter region (*TrpA1* ^*KI-GAL4*^) [33]. In the wake of these results, we asked whether the effects of dTRPA1 on behaviour are through the circadian neuronal subsets that overlap with the *dTRPA1* ^*SH*^ *-GAL4* driver. We modified dTRPA1 expression levels using *UAS-dTRPA1* in the circadian neurons with different drivers—*pdf-GAL4* driver [31] which specifically targets only the LN_v_ and *cry-GAL4-39* driver [32] which targets LN_v_ and LN_d_ and DNs (Fig 4A). It is known from previous studies that *UAS-hid* mediated neuronal ablation under *Pdf-GAL4* and *cry-GAL4-39* removes all PDF-positive LN_v_ [31,43] and all CRY^+^ neurons [7] respectively. Neuronal ablation of LN_v_ (with *Pdf-GAL4*) or CRY^+^ neurons (with *cry-GAL4-39*) did not abolish A-peak in flies (Fig 5A, left panels; Fig 5B) suggesting that circadian neurons expressing dTRPA1 have little influence on occurrence of A-peak under gradual environmental cycles. Increasing dTRPA1 expression either in the LN_v_ alone or in CRY^+^ circadian neurons has no effect on occurrence of A-peak (Fig 5A, right panels; Fig 5B). As a further verification of this finding, when dTRPA1 levels are overexpressed in dTRPA1 neurons except in CRY^+^ neurons (*dTRPA1* ^*SH*^ *-GAL4/cry-GAL80 < UAS-dTRPA1*) A-peak is enhanced in these flies (Fig 5C and 5D), similar to *dTRPA1* ^*oex*^ flies under L_r_+T_r32_ (out of phase), DD+T_r32_ and LL+T_r32_ (Figs 2–4). This suggests that non-CRY neurons under *dTRPA1* ^*SH*^ *-GAL4* are the predominant modulators of enhanced A-peak activity levels. Although some degree of overlap exists between the *dTRPA1* ^*SH*^ *-GAL4* and circadian pacemaker neurons as suggested by anatomical evidence [42], we show that dTRPA1 in these CRY^+^ circadian neurons (fifth s-LN_v_ and LN_d_) do not play a major role in mediating occurrence of dTRPA1-dependent A-peak. We, however, cannot rule out the possibility that CRY negative, dTRPA1^+^ neuronal subset does not include other circadian neurons. Thus, we propose that the CRY negative, dTRPA1^+^ neurons are the primary modulators of afternoon activity.

### Thermosensitive isoform dTRPA1-A can rescue the occurrence of A-peak in a dTRPA1 null background

A recent study has revealed that the *dTRPA1* gene codes for four alternatively spliced isoforms—dTRPA1-A-D, out of which B and C isoforms are not temperature-responsive [27]. dTRPA1-A isoform responds to temperature range 24–29°C whereas dTRPA1-D activates at 34°C [27]. Since the isoforms of dTRPA1 are activated at distinct temperature ranges and dTRPA1-dependent A-peak in our experiments is seen around 32°C (above the temperature threshold for dTRPA1-A, but below the threshold for dTRPA1-D), we aimed to distinguish which of the temperature sensitive isoforms of dTRPA1 mediates the occurrence of A-peak. We overexpressed temperature-sensitive isoform, *UAS-dTRPA1-A* and *UAS-dTRPA1-D* under *dTRPA1* ^*SH*^ *-GAL4* and subjected the flies to L_r_+T_r32_, a regime which we demonstrate adequately mimics conditions that elicit the A-peak. Overexpressing dTRPA1-A isoform or the higher temperature threshold dTRPA1-D isoform in dTRPA1^+^ neurons induces an A-peak but the amplitude of A-peak is not different from their respective parental controls (Fig 6A and 6C, left panels). Next, we rescued dTRPA1 expression in neurons targeted by *dTRPA1* ^*SH*^ *-GAL4* in the null *dTRPA1* ^*ins*^ background by either expressing *UAS-dTRPA1-A* or *UAS-dTRPA1-D*. Rescue using *UAS-dTRPA1-A* isoform caused an enhanced A-peak in the *dTRPA1* ^*ins*^ background (Fig 6B, left panel; Fig 6C, right panel-one-way ANOVA, *F*_(1,142)_ = 66.9, *p* < 0.05). Expressing *UAS-dTRPA1-D* isoform in the null background shows a small bout of activity similar to its GAL4 control in a null background (Fig 6B, right panel, compare green and grey curves). In this assay, a small fraction of all parental controls in null background displayed A-peak (Fig 6B grey and black curves, S2 Table). We conclude that the dTRPA1-A isoform is critical for the induction of A-peak under the simulated SN conditions since restoring functional isoform A in neurons targeted by *dTRPA1* ^*SH*^ *-GAL4* in a null background is sufficient to elicit A-peak.

## Discussion

Insects exhibit many physiological adaptations to high temperature and low humidity in nature. In addition to modulating respiration and metabolism, behavioural modulation is an important adaptive response that requires temperature sensing. dTRPA1 ion channels have been shown to be essential for thermosensation in nematodes, flies and mammals including humans [29]. Several recent studies show that under semi-natural conditions, flies exhibit an additional peak of activity during mid-day that could be a reflection of an ‘escape response’ or an ‘environmentally modulated circadian’ response to hot daytime temperatures [10–12]. Although rhythmic regulation of activity in fruit flies under natural conditions remains far from understood, recent studies suggest that the three activity peaks (M, A and E) are modulated by both environmental factors and circadian clocks [10,12], while other studies including our own posit that non-clock mechanisms can also induce the A-peak [11,14]. In nature, light and temperature would be expected to modulate activity levels [13] in a complex fashion. Here we demonstrate a temperature-dependent mechanism for the A-peak response exhibited by flies under SN. Our study shows that the A-peak is a temperature-dependent mid-day response mediated via dTRPA1. Previously we used visual observations to examine the behaviour of flies under SN placed in different spatial arenas including glass tubes used for measuring locomotion and also larger arenas such as petri-dishes. These studies suggested that flies seek shaded regions in the small glass tubes and that when they are provided larger arenas, they do not show heightened A-activity under similar environmental cycles. One major and perhaps critical distinction between our previous study [11] and those of Vanin et al. [10] and Menegazzi et al. [12] which report very high levels of afternoon activity (compared to De et al. [11]) is that the average temperature in all the assays reported by De et al was never above 29°C. Hence, qualitatively, the type of activity exhibited could be different in the two conditions. Later studies from our lab have shown that both light and temperature can induce A-peak [13]. Based on 12 independent assays conducted over 1.5 years we showed that for *D*. *melanogaster* under SN, the occurrence of A-peak was positively correlated with both average daytime temperature (R^2^ = 0.13) and light (R^2^ = 0.37). Furthermore, we subjected flies to simulations of natural light and / or temperature cycles in the laboratory and showed that sufficiently high maxima of either light or temperature alone can elicit A-peak. This is verified by our current results which show that under SN, if the light and temperature peaks are clearly separated in time, two distinct A-peaks are produced, one in response to L_max_ and the other in response to T_max_. Since the T_max_ induced A-peak can be elicited at any phase of daytime, it further confirms that it is independent of circadian clock control (S3B Fig). Based on our past results and our new data our current view is that when peak daytime temperatures are relatively low, flies will tend to seek shade whereas when temperatures rise above (~29°C) or if light intensities are sufficiently high (~ 3000 lux) then flies show more intense locomotion. dTRPA1 is critical for this temperature sensitive locomotor activity.

In most cases when temperatures rise beyond a certain threshold, almost 100% wild type flies exhibit A-peak (Table 1), however, in some conditions, for example, L_r_+T_r32_ out-of-phase, only 61% of control *UAS-TRPA1* flies show A-peak. This suggests that either the thermal sensation is not conveyed to those cells that eventually control motor activity, or that the neuronal circuits in those flies require to cross a higher threshold in order to produce this behaviour. Under experimental regimes such as DD+SN, DD+T_r32_ and LL+T_r32_, wild type flies display a clear A-peak whereas dTRPA1 null flies do not exhibit an A-peak. The above results demonstrates that gradual temperature cycles with adequate contrast in temperature range and with a peak temperature around 32°C is sufficient to elicit A-peak response and supports our hypothesis that dTRPA1 mediated A-peak is temperature dependent. However, ablating majority of neurons under *dTRPA1* ^*SH*^ *-GAL4* driver does not completely abolish occurrence of A-peak under SN, DD+SN or simulated SN regimes in the laboratory. This result can be explained most parsimoniously by the narrow expression pattern of *dTRPA1* ^*SH*^ *-GAL4* driver that may not encompass the full range of cells in which dTRPA1 is natively expressed. Alternatively it is also likely that gradual changes in temperature profile under SN or simulated SN laboratory protocols activates other thermoreceptors that function in temperature ranges adjacent to dTRPA1 activation. Under such conditions, even flies with attenuated dTRPA1 function are likely to be able to respond to high temperatures during mid-day. This phenomenon can also be seen in *dTRPA1* ^*ins*^ null flies that show short bouts of activity in response to temperature shifts under LL+T_r32_ (Fig 4A) and more clearly under DD+T_r32_ (Fig 3A) while not responding to temperature shifts from 28–30°C or 30–32°C.

The A-peak seen under SN or gradually changing light and temperature cycles in the laboratory is likely to be a reflection of the fly’s sensation of adverse warm temperatures and its attempts to escape from it. In our studies, flies lacking dTRPA1 are perhaps unable to perceive or respond to temperature changes beyond 28°C and consequently do not exhibit the A-peak. Over-expressing dTRPA1 in a small subset of dTRPA1^+^ neurons renders the flies acutely thermosensitive. This provides direct evidence for the involvement of dTRPA1 in mediating the A-peak. This specific modification also causes a delay in the phase of the E-peak. Since E-peak is not a mere response to temperature, but a circadian clock-controlled activity, and flies are likely to compensate (homeostatically) for the increased activity of the afternoon with a depression in activity in the evening, we speculate that both factors come into play in modifying E-peak phase in these flies. Chronic activation of these neurons (using *UAS-NaChBac*) did not amplify the A-peak suggesting compensatory or buffering mechanisms in the circuits that regulate the amplitude of this peak. This likely occurs by modulation of downstream neurons that receive multiple inputs from temperature and circadian circuits and future studies may reveal the pathways by which thermosensors influence rhythmic locomotor activity.

Further, although dTRPA1 expression overlaps with circadian neurons, we show that the CRY negative dTRPA1^+^ neurons are the primary drivers of the A-peak under gradual environmental cycles. We are able to distinguish that the non-CRY expressing dTRPA1 neurons modulate enhanced A-peak under gradual light and temperature cycles thus eliminating the role for CRY^+^ fifth s-LN_v_ and LN_d_ that overlap with *dTRPA1* ^*SH*^ *-GAL4* targeted neurons. Thus while complete lack of functional dTRPA1 ion channels abolishes occurrence of A-peak under SN and laboratory simulated SN conditions, a small number of CRY negative dTRPA1^+^ neurons are sufficient to modulate afternoon activity. Other TRP ion channels like *painless* and *pyrexia* function at temperature ranges different from those used in our study. *Painless* [44] activates directly in the noxious range (around 40°C) and *pyrexia* which either directly activates in the noxious temperature range [45] or through indirect activation functions at lower temperature range (16–20°C) [46]. While dTRPA1 ion channels have been implicated in one study as mediating thermal nociception [21], it has been mostly studied in the context of thermotaxis at a much lower temperature range (24–29°C) [19,22–27]. Besides the direct thermal activation of dTRPA1, it has also been suggested that dTRPA1 channels can be indirectly activated by *norpA* encoded PLC pathways to function at 19°C to mediate larval thermotaxis [23]. dTRPA1 is an ideal candidate for mediating behavioural changes in response to a range of temperatures more likely to be encountered in the natural habitat of flies where they exhibit diurnal rhythms.

Several attempts have been made to determine the activation range of dTRPA1 ion channels. Based on the methodology adopted, the results vary. Recordings from heterologous system put dTRPA1 activation threshold between 24–29°C [28] whereas Ca^+2^ imaging from AC neurons [19,26] and NMJ recordings [19] show that dTRPA1 neurons respond to temperatures above 25°C. Further, the two temperature-responsive isoforms dTRPA1-A and dTRPA1-D have activation ranges of 24–29°C and 34°C respectively [27]. We find that rescue of dTRPA1 function in *dTRPA1* ^*ins*^ background with A isoform is sufficient to rescue the induction of A-peak, whereas D isoform that is activated at a temperature range slightly above the T_max_ of our experimental regime does not rescue the A-peak. We cannot rule out the possibility that dTRPA1 isoforms are differentially expressed under the *dTRPA1* ^*SH*^ *-GAL4* driver. However, since the activation range of the dTRPA1-D isoform is higher than T_max_ used in our experiment we think it is unlikely that the expression levels of the isoforms affect the behavioural phenotype. The above results reiterate our findings that A-peak is predominantly a temperature driven phenomenon and is dependent on dTRPA1-A. Under SN or even DD+SN, possibly several factors including temperature contribute to the generation of the A-peak. Of these, dTRPA1 seems to be critical, however, the cells in which dTRPA1 must be expressed is not revealed by the *dTRPA1* ^*SH*^ *-GAL4* driver (S2 Fig). It is clear however that its expression in the cells targeted by this driver is sufficient for the behaviour (Fig 6). Taken together, we speculate that among the dTRPA1 expressing cells, those targeted by the *dTRPA1* ^*SH*^ *-GAL4* alone can generate a temperature dependent peak. However, eliminating them does not prevent A-peak since there are other cells outside of the target of this driver that are sufficient to elicit the A-peak.

Based on our present understanding of dTRPA1 functions we propose that the conduction of thermal input from the CRY negative, thermosensory dTRPA1 neurons is crucial for regulating the mid-day behavioural responses to temperature. Under semi-natural conditions when gradually increasing temperature reaches a mid-day peak above 28°C, dTRPA1^+^ neurons likely activate downstream neurons that enhance locomotor activity, facilitating escape from stressful conditions. On the other hand, lack of dTRPA1 renders flies unable to sense warm temperatures and hence dTRPA1 null flies do not exhibit A-peak. Therefore, we posit that on hot days, dTRPA1 mediated increased locomotion may enable flies to escape stressfully hot conditions.

## Supporting Information

S1 Fig Verification of GAL80 mediated suppression of GFP expression with dTRPA1^SH^-GAL4 in Drosophila adult brains. (A, left) GFP expression was absent from both LN_v_ and LN_d_ cell clusters when *cry-GAL80* was combined with *dTRPA1* ^*SH*^ *-GAL4* driving *UAS-2xeGFP*. (A, right) GFP labelled dTRPA1^+^ neurons were seen overlapping with two LN_d_ but no overlap was seen in LN_v_ cluster when *pdf-GAL80* was used with *dTRPA1* ^*sh*^ *-GAL4*. Scale bars = 20μm (B) No GFP labelled dTRPA1^+^ neurons were detected when *dTRPA1* ^*SH*^ *-GAL4* targeted neurons were ablated using *UAS-hid* in six out of seven brains. The only brain with a GFP positive cell is shown here. Scale bar = 50μm.
(EPS)

S2 Fig Average activity profiles of flies with altered dTRPA1 expression under semi-natural condition. (A, left) Flies with dTRPA1 ablated neurons (blue curve) also responded with an A-peak under SN with very high light intensity (~2100 lux), indicating that cells outside the target of *dTRPA1* ^*SH*^ *-GAL4* driver can mediate the occurrence of A-peak. (A, right) Flies with dTRPA1 over-expression (*dTRPA1* ^*oex*^- blue curve) also show A-peaks under SN similar to their controls. (B) Similar to SN conditions, under DD+SN, (left) neuronal ablation of dTRPA1 neurons did not abolish the occurrence of the A-peak. (B, right) dTRPA1 over-expression caused dramatic increase in the A-peak (arrow, right panel) under DD+SN and also delayed evening peak (arrowhead). All other details same as Fig 1.
(EPS)

S3 Fig A-peak occurrence depends on dTRPA1 levels under simulated natural conditions in the laboratory. (A, top) Average actograms of flies exposed to L_r_+T_r32_ for first eight days during which T_max_ was 32°C for one hour (orange shading) followed by L_r_+T_r28_ for nine days when T_max_ was 28°C for one hour. (Top row) *w* ^*1118*^ flies show mid-day peak only during L_r_+T_r32_ whereas *dTRPA1* ^*ins*^ do not show a mid-day peak in either simulated T_r_ conditions. (A, bottom) Flies with increased dTRPA1 levels- *dTRPA1* ^*oex*^ flies, also show A-peak similar to their controls only under L_r_+T_r32_. (B) Induction of A-peak at different times of day under LD (12:12) cycles. Flies were subjected to LD/ 21°C for 3 days before imposing a gradual temperature cycles with T_max_ at 6 hr after Lights-ON during LD+T_r1_ and at 3hr after Lights-ON during LD+T_r2_. Wild type *w* ^*1118*^ flies showed A-peak coinciding with the timing of T_max_ (arrows) whereas *dTRPA1* ^*ins*^ flies did not show any A-peak. Yellow shaded area represents light phase of LD cycles.
(EPS)

S4 Fig Hyperexciting dTRPA1 neurons also elicits A-peak. Average activity profiles of flies driving *UAS-NaChBac1* in neurons targeted by *dTRPA1* ^*SH*^ *-GAL4* in simulated temperature cycles under constant darkness (DD+T_r32_). Flies with hyperexcited dTRPA1^+^ neurons exhibit an A-peak similar to control flies and have a slightly delayed evening peak (arrowhead).
(EPS)

S1 Table Details of experimental regimes used in the study. (DOC)

S2 Table Percentage of flies from different genotypes displaying A-peak under modified laboratory simulated SN regimes. (DOCX)
