# Supplementary material for: dTRPA1 Modulates Afternoon Peak of Activity of Fruit Flies Drosophila melanogaster
Source: PLoS One. 2015 Jul 30;10(7):e0134213. doi: 10.1371/journal.pone.0134213 (PMC4520709; doi:10.1371/journal.pone.0134213)
Supplement: S1 Table — (DOC) [file pone.0134213.s005.doc]

| **Regime** | **Light condition** | **Temperature condition** | **Phase difference** |
| --- | --- | --- | --- |
| L_r_+T_r32_ (In-phase) | Gradually changing (ramped) light intensity cycles reaching a peak of 1800 lux | Gradually changing (ramped) temperature from 17 ^o^C to 32 ^o^C | 0 hr (In-phase) |
| L_r_+T_r32_ (Out of-phase) | Gradually changing (ramped) light intensity cycles reaching a peak of 1800 lux | Gradually changing (ramped) temperature from 17 ^o^C to 32 ^o^C | 3hr (Light peak 3 hr prior to temperature peak) |
| L_r_+T_r28_ (In-phase) | Gradually changing (ramped) light intensity cycles reaching a peak of 1800 lux | Gradually changing (ramped) temperature from 17 ^o^C to 28 ^o^C | 0 hr (In-phase) |
| LL_100_+T_r32_ | Constant light (LL = 100 lux) | Gradually changing (ramped) temperature from 17 ^o^C to 32 ^o^C | NA |
| DD+T_r32_ | Constant darkness (DD = 0 lux) | Gradually changing (ramped) temperature from 17 ^o^C to 32 ^o^C | NA |
| LD+T_21_ | LD (12hr :12hr) | Constant temperature of 21 ^o^C | NA |
| LD+T_r1_ | LD (12hr :12hr) | Gradually changing (ramped) temperature from 17 ^o^C to 32 ^o^C with T_max_ at 6 hr after Lights-ON (16:00 hrs) | NA |
| LD+T_r2_ | LD (12hr :12hr) | Gradually changing (ramped) temperature from 17 ^o^C to 32 ^o^C with T_max_ at 3 hr after Lights-ON (13:00 hrs) | NA |

S1 Table. Details of laboratory experimental regimes.
