# Supplementary material for: dTRPA1 Modulates Afternoon Peak of Activity of Fruit Flies Drosophila melanogaster
Source: PLoS One. 2015 Jul 30;10(7):e0134213. doi: 10.1371/journal.pone.0134213 (PMC4520709; doi:10.1371/journal.pone.0134213)
Supplement: S2 Table — (DOCX) [file pone.0134213.s006.docx]

| **Figure No and Regime** | **Genotypes** | **N** | **% flies exhibiting A-peak** |
| --- | --- | --- | --- |
| **3A. DD+T_r32_** | *w^1118^* | 30 | 100 |
|  | *dTRPA1^ins^* | 31 | 22.5 |
|  | *dTRPA1^SH^-GAL4/ UAS dTRPA1* | 32 | 100 |
|  | *dTRPA1^SH^-GAL4/ +* | 28 | 96.4 |
|  | *UAS dTRPA1/ +* | 31 | 90.3 |
|  | *TrpA1^KI-GAL4^* | 24 | 16.7 |
|  | *TrpA1 ^KI-GAL4/+^* | 32 | 96.7 |
|  | | | |
| **4A. LL+T_r32_** | *w^1118^* | 28 | 100 |
|  | *dTRPA1^ins^* | 24 | 0 |
|  | *dTRPA1^SH^-GAL4/ UAS dTRPA1* | 29 | 100 |
|  | *dTRPA1^SH^-GAL4/ +* | 28 | 100 |
|  | *UAS dTRPA1/ +* | 27 | 100 |
|  | | | |
| **5A. L_r_+T_r32_** | *Pdf -GAL4/ UAS dTRPA1* | 29 | 100 |
|  | *Pdf -GAL4/ UAS hid* | 18 | 100 |
|  | *Pdf -GAL4/ +* | 29 | 100 |
|  | *cry-GAL4-39/ UAS dTRPA1* | 30 | 100 |
|  | *cry-GAL4-39/ UAS hid* | 21 | 100 |
|  | *cry-GAL4-39/ +* | 27 | 100 |
|  | *UAS dTRPA1/ +* | 30 | 100 |
|  | *UAS hid/ +* | 28 | 100 |
| **5C. L_r_+T_r32_** | *dTRPA1^SH^-GAL4;cry –GAL80/ UAS dTRPA1* | 8 | 100 |
|  | *dTRPA1^SH^-GAL4;cry –GAL80/ +* | 29 | 100 |
|  | *UAS dTRPA1/ +* | 31 | 100 |
|  |  |  |  |
| **6A. L_r_+T_r32_** | *dTRPA1^SH^-GAL4 / UAS dTRPA1-A* | 16 | 100 |
|  | *dTRPA1^SH^-GAL4 / UAS dTRPA1-D* | 15 | 100 |
|  | *dTRPA1^SH^-GAL4 / +* | 16 | 100 |
|  | *UAS dTRPA1-A/ +* | 16 | 87.5 |
|  | *UAS dTRPA1-D/ +* | 16 | 87.5 |
| **6B. L_r_+T_r32_** | *dTRPA1^SH^-GAL4 / UAS dTRPA1-A; dTRPA1^ins^* | 28 | 100 |
|  | *dTRPA1^SH^-GAL4 / UAS dTRPA1-D; dTRPA1^ins^* | 30 | 50 |
|  | *dTRPA1^SH^-GAL4 / +; dTRPA1^ins^* | 29 | 41.3 |
|  | *UAS dTRPA1-A/ +; dTRPA1^ins^* | 29 | 24.1 |
|  | *UAS dTRPA1-D/ +; dTRPA1^ins^* | 31 | 19.3 |

**S2 Table:** Percentage of flies from different genotypes displaying A-peak under laboratory simulated SN regimes. For details regarding genotypes, please refer to the Methods section.
